# INSIGHT: A Phase III Trial of Ripretinib Versus Sunitinib in Patients with Advanced GIST with *KIT* Exon 11 and Exon 17/18 Mutations Who Were Previously Treated with Imatinib

**DOI:** 10.1245/s10434-024-16853-x

**Published:** 2025-02-05

**Authors:** Carolina Larrain, Tracey Pu, Paulina Cox, Kam Sprott, Andrew M. Blakely, Sebastian Bauer, Suzanne George

**Affiliations:** 1 Surgical Oncology Program, Center for Cancer Research, National Cancer Institute, National Institutes of Health, Bethesda, MD USA; 2 Deciphera Pharmaceuticals, LLC, Waltham, MA USA; 3 Department of Medical Oncology, Sarcoma Center, West German Cancer Center, University Hospital Essen, University Duisburg-Essen, Essen, Germany; 4 German Cancer Consortium (DKTK), Partner Site University Hospital Essen, Essen, Germany; 5 Dana-Farber Cancer Institute, Boston, MA USA

## Methods

See the electronic supplementary materials.

## Clinical Context

Gastrointestinal stromal tumors (GISTs) are the most common tumor of mesenchymal origin in the gastrointestinal tract, with an incidence of 10–15 cases per million per year.^1^ The majority (approximately 85%) of GISTs are characterized by gain-of-function mutations in the receptor tyrosine kinases encoded by *KIT* or *PDGFRA*.^2^ The most common mutational drivers of GIST are *KIT* exon 11 mutations.^3^ These mutations are generally associated with a favorable response to imatinib, a tyrosine kinase inhibitor (TKI) that is the mainstay of medical therapy for advanced disease. However, tumors may develop resistance to imatinib through the accumulation of secondary mutations, such as those within the KIT ATP-binding pocket (exons 13 and 14) or activation loop (exons 17 and 18).^4^ This acquired resistance may necessitate transition to sequential TKIs, such as sunitinib (second-line) and regorafenib (third-line). Ripretinib is a fourth-line switch-control TKI designed to broadly inhibit KIT and PDGFRA with a favorable safety profile.^5^

The recently reported INTRIGUE phase III trial examined ripretinib versus sunitinib in patients with advanced GIST previously treated with imatinib. The primary endpoint of superior progression-free survival (PFS) with ripretinib over sunitinib was not met; however, ripretinib demonstrated comparable efficacy to sunitinib with a favorable safety profile.^6^ Interestingly, a subsequent exploratory analysis of the data demonstrated that patients with primary *KIT* exon 11 mutations and secondary mutations exclusively in exon 17/18 detected in ctDNA had a significant improvement in median PFS when treated with ripretinib compared with sunitinib (14.2 vs. 1.5 months; nominal *p *< 0.0001). Conversely, patients with primary *KIT* exon 11 and secondary exon 13/14 mutations detected in ctDNA had significantly improved outcomes when treated with sunitinib (median PFS 15.0 vs. 4.0 months; nominal *p *= 0.0005).^7^ The INSIGHT trial, run by co-principal investigators Sebastian Bauer, MD, and Suzanne George, MD, aims to further explore the favorable response to ripretinib in patients with primary *KIT* exon 11 and secondary *KIT* exon 17/18 mutations. This international, multicenter, phase III, randomized controlled trial will consist of two arms comparing second-line sunitinib versus ripretinib in patients with advanced GIST previously treated with imatinib who harbor a primary *KIT* exon 11 mutation and secondary mutations exclusively in exon 17/18.^8^ The primary endpoint of the trial is PFS measured by independent radiologic review per modified Response Evaluation Criteria in Solid Tumors version 1.1 (mRECIST v1.1); secondary endpoints include objective response rate and overall survival. Patients must have a histologic diagnosis of GIST with mutational status confirmed by circulating tumor DNA (ctDNA) at prescreening.

## Investigator Insights

Despite differences in mutational profiles, the treatment algorithm for *KIT*-mutant GIST follows a standard TKI sequence.^9^ Once patients are deemed resistant to first-line therapy, they are transitioned from imatinib to sunitinib (second-line), regorafenib (third-line), and ripretinib (fourth-line), following the original order in which these agents received US FDA approval. Notably, the trials that led to the approval of these agents did not account for mutational status and included patients with diverse GIST mutational profiles, likely including molecular subtypes now known to be resistant to TKI therapy.^10,11,5^ Data on how mutational status should affect selection of TKI therapy, specifically within *KIT*-mutant GIST, are lacking. The INSIGHT trial aims to further explore this point by examining how primary *KIT* exon 11 and secondary *KIT* exon 17/18 mutations affect response to sunitinib versus ripretinib. Positive results from INSIGHT could change how second-line therapy is selected, favoring an individualized approach based on mutational status.

Advancements in TKI therapy will likewise have important implications on the surgical management of advanced GISTs. While the role of metastasectomy remains to be fully defined, some patients with stable or responsive disease on TKI therapy have improved outcomes after metastasectomy compared with patients who have TKI-resistant disease. ^12,13^ Furthermore, improved response to TKI therapy may convert lesions deemed unresectable to a resectable status, expanding the pool of surgical candidates—an important implication, as complete surgical resection remains the only curative option for GIST.

The INSIGHT trial also aims to further explore the role of ctDNA, with the goal of providing a more comprehensive method of assessing the genetic composition of tumors. TKI-resistant GISTs have heterogeneous mutational profiles, with multiple secondary mutations developing between metastases, or even within the same lesion.^4^ Current biopsy techniques are limited and can only provide a partial mutational assessment based on which lesions are selected for biopsy. Analyzing ctDNA may provide a less invasive and more comprehensive way of assessing mutational status, making the detection of secondary resistance mutations more feasible and reflecting the entire disease burden across multiple metastatic sites.^14^ Overall, this trial is an important step in shifting the treatment paradigm for *KIT-*mutant GIST, improving assessment of mutational status, and exploring targeted second-line therapy based on a patient’s individual mutational profile. 
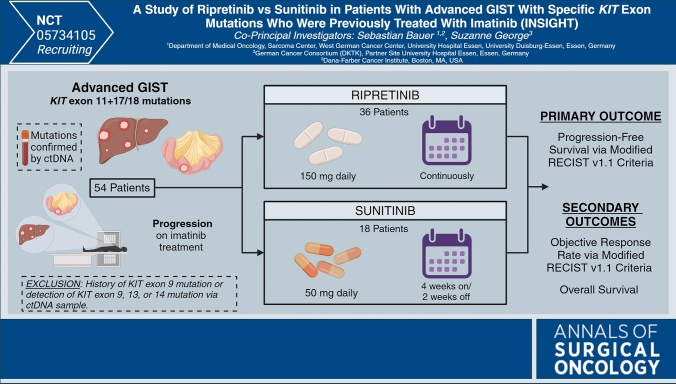


## Supplementary Information

Below is the link to the electronic supplementary material.

Supplementary file1 (DOCX 523 KB)
