# Supplementary material for: INSIGHT: A Phase III Trial of Ripretinib Versus Sunitinib in Patients with Advanced GIST with KIT Exon 11 and Exon 17/18 Mutations Who Were Previously Treated with Imatinib
Source: Ann Surg Oncol. 2025 Feb 5;32(5):3065–7. doi: 10.1245/s10434-024-16853-x (PMC11976832; doi:10.1245/s10434-024-16853-x)
Supplement: Supplementary file 1 — Supplementary file1 (DOCX 523 KB) [file 10434_2024_16853_MOESM1_ESM.docx]

**SUPPLEMENTAL METHODS**

ClinicalTrials.gov was queried for trials using the term “gastrointestinal stromal tumor.” The resulting trials were then filtered for actively recruiting studies which are interventional in nature, leaving a selected group of studies available. From these, studies were selected based on a diversity of study topics and pertinence to the practice of surgical oncology (Supplemental Fig. 1).

The primary investigators were individually contacted via email for their consent in participating in this series and were asked a set of standardized questions from which the information provided was editorialized into the “Investigator Insights” section.

All figures were created using BioRender.com.

**SUPPLEMENTAL FIGURE 1.**

**
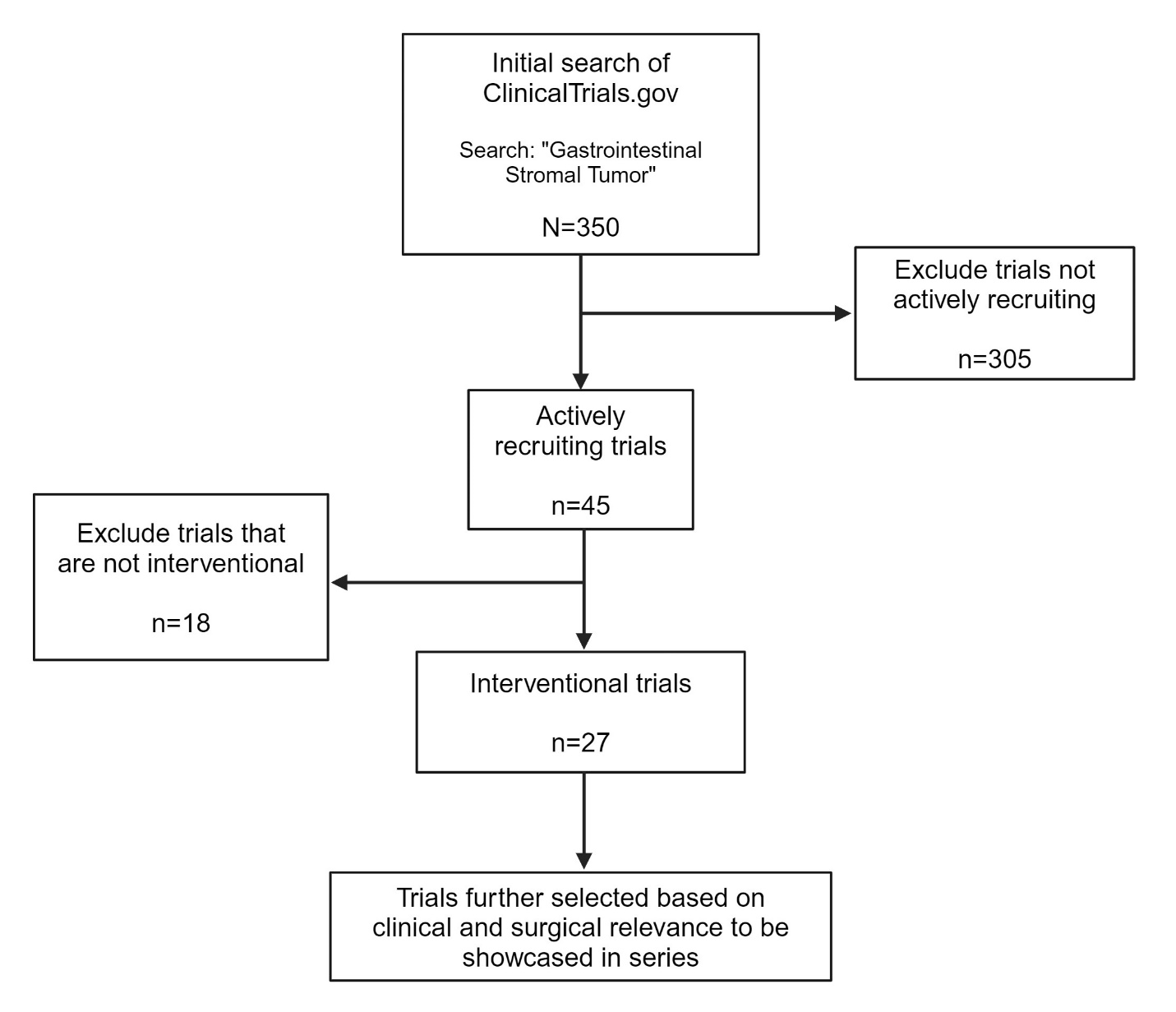
**
